# A phylogenetic analysis of numeral anchor choice in New Guinea and lowland South America

**DOI:** 10.1098/rstb.2024.0222

**Published:** 2025-10-20

**Authors:** Annemarie Verkerk, Elisa Castillo Atehortua, Christoph Rzymski

**Affiliations:** ^1^ Universitat des Saarlandes, Saarbrücken 66123, Germany; ^2^ Department of Linguistic and Cultural Evolution, Max-Planck-Institute for Evolutionary Anthropology, Leipzig 04103, Germany

**Keywords:** linguistics, language change, phylogenetics, language contact, indigenous languages, numeral anchor

## Abstract

Most of the world’s languages have numeral systems with a decimal base, but in some areas, such systems are rare. This article focuses on languages with either restricted numeral systems or systems featuring compositional anchors that are *not* bases, examining how they are used in the creation of non-atomic numerals. We investigate anchor choice from an explicitly diachronic perspective by modelling it on phylogenetic trees for language families from two areas: northern lowland South America (Arawakan, Pano-Tacanan, Tucanoan and Tupian) and Papuan languages of New Guinea (Nuclear Torricelli, Nuclear Trans New Guinea, Ramu and Sepik). We find languages with restricted numeral systems lacking anchors, as well as systems with anchor 2 or 5. Phylogenetic analyses suggest that the proto-languages of these families likely had restricted or anchor 2 systems, with shifts to anchor 5 occurring in specific groups, indicating some time depth, although statistical power is limited by small family sizes. Further research should study linguistic systems alongside cultural counting practices, reconstruct both linguistic forms and cultural practices and model the impact of contact, especially given the global dominance of decimal systems.

This article is part of the theme issue ‘A solid base for scaling up: the structure of numeration systems’.

## 1. Introduction

For the billions of us that speak a language with a decimal numeral system, there is something special about the number 10. We often measure time in blocks of 10 units (think of the phrase ‘take ten’ or countdowns starting with 10); in scoring in gymnastics or reviews, 10 represents perfection or completeness; birthdays and wedding anniversaries that are multiples of 10 are considered special. Sarton [1] conveys this view when he writes (p. 581): ‘Thus the decimal system was often (and still is) unnecessarily complicated with quinary, duodecimal, vigesimal and sexagesimal ideas. […] Every language contains similar oddities which introduce unnecessary complications but do not jeopardize nor even conceal the [decimal, AU] number system.’ Sarton [1] and others (such as [2]) believe that the reason we find decimal systems again and again throughout the world is rooted in the make-up of what he calls our ‘natural counting machine’: fingers (and toes). Indeed, even the less common alternatives to the decimal system—vigesimal (base 20), and the (contested^1^) case of quinary (base 5)—can be explained as ultimately leading back to situations where people count on their fingers and toes (see Chrisomalis [5], pp. 539−542] for a different view).

Looking at numeral bases today, we indeed find that the decimal base is by far the most common one: Comrie [6] reports that 125 languages out of a sample of 196 languages have decimal systems (64%), and in Grambank v. 1.0 [7], 1234 out of 2189 investigated languages have decimal systems (56%). Grambank [7] also asks about (compositional) bases 5 and 20, the former being attested in 386 languages (18%) and the latter in 271 (12%). However, the distribution of decimal bases across the world is not equal. There are areas that lack a decimal base, most notably Australia, the island of New Guinea, and in parts of South America (see also Epps *et al.* [8]). ‘Quinary’ bases are found mostly in sub-Saharan Africa and the Americas, while vigesimal systems are somewhat common in Meso-America and pockets of Eurasia.

This distribution raises the question of how decimal systems have become so frequent and widespread, and how they ‘interact’, both diachronically and cognitively, with other bases and compositional anchors more broadly. In many language families, a decimal system can be reconstructed for the proto-language. This is true for Austrosiatic (even though Sidwell [9] considers it a late change from a so-called restricted numeral system), Austronesian [10, p. 278], Bantu (but not the rest of Niger-Congo [11, p. 26]), Indo-European (see [12] and sources in Bauer [13], who herself argues for a vigesimal proto-system) and Uralic [14], to mention a few families. In many of these, base 10 has been around for millennia and can hence be considered very stable. This may be pointed out specifically for the Pacific context: Schapper and Hammarström [15] show that despite contact-induced innovations, eastern Malayo-Polynesian (Austronesian) languages rarely lose their inherited decimal base altogether.

Besides having another base, an important alternative to a decimal base is not having a base at all. This is the case when a language has a restrictive or unproductive numeral system (20 out of 196 languages in Comrie’s sample [6]). Comrie [6] defines a restrictive system as ‘a numeral system that does not effectively go above around 20’. As mentioned above, Australia is one of the places where decimal systems are not found, and that correlates with many Pama-Nyungan languages having restricted numeral systems [16,17]. Most typical is an upper limit of 3 or 4, but a few Pama-Nyungan languages have numerals up to 10 or even 20. In short, variation in numeral base and system is captured not just in the type of base, but also in whether a base is used at all—and possibly whether *numerals* are present at all [18]. Aside from Pama-Nyungan in Australia [16,17], the areas where decimal bases are mostly absent have not been investigated in great detail. The only exception is the large-scale survey by Epps *et al.* [8] of numeral systems from languages spoken by hunter–gatherer groups, who have traditionally been said to have restricted systems. They find that, among other areas, Amazonia is characterized by languages without true numerals and that multiple aspects of numeral systems (including bases) are areal.

In this article, we focus on two relevant areas lacking decimal systems: the island of New Guinea and northern lowland South America. In order to capture the different types of compositional structure in a more nuanced manner, theorists across disciplines (Chrisomalis [2], Barlow [3] and Pelland [4]) are now advocating a new terminological framework that we also adopt. Accordingly, a *base* is a special compositional element within a numeral system; Chrisomalis [2] writes ‘[f]or the purposes of this analysis, a base of a numeral system B is a number in a system that is marked by a shift in counting unit, and whose powers are also highlighted by shifts in counting units. […] Other numbers that are actively used in the construction of composite numerals, but whose powers do not mark a structural shift, will be called anchors.’ Pelland [4] distinguishes several anchors, and many of the anchors we encounter in this article could be called *sporadic anchors*, defined by Pelland as ‘[a] number that is used to produce less than half of the non-atomic numerals within a counting cycle that starts at 1 and ends at the largest non-hapax anchor of the system for non-base systems, or between two powers of the base for base systems’ [4]). We stick to the term *anchor* to describe compositional numerals, as it allows us ‘to talk about any number that is used to compose non-atomic numerals’ (Pelland [4]) without having to posit that such a numeral is a base.^2^

In our coding, we use the presence of an anchor in a system as the criterion for categorizing that system, and code for restrictive systems (which lack any such anchor) as well as various anchors; the latter according to their atomic value: anchor 2, 4, 5, 6, 10 and 20. For example, in Matsés (Pano-Tacanan), *daid daid* means ‘four’, and consists of the repetition of the word meaning ‘two, lit. to increase in number’ (Fleck [20]). Here, *daid* ‘two’ is used as an anchor forming the non-atomic numeral 4. Note that systems occasionally have multiple anchors, we take the use of any unique, atomic form-meaning combination (such as *daid*) in the formation of non-atomic numerals as indicative of anchor status. We find an example of this in Capanahua (Pano-Tacanan; Loos [21]) with an anchor 2 and an anchor 5, which we code as having compositional forms 1, 2, 2 + 1, 2 + 2, 5, 5 + 1, 5 + 2, 5 + 3 (3 borrowed from Quechua), 5 + 4 (4 borrowed from Quechua), 5 + 5, 5 + 5 + 1, … 5 + 5 + 5 + 5. We are interested in all attested anchors and especially their diachronic emergence and loss, not just the dynamics of the smallest anchor. Hence, we use terms such as ‘anchor 2 system’ and ‘anchor 5 system’ as shorthands for ‘system with 2 as only anchor’, but *also* for ‘system with 2 as the smallest anchor’ and ‘system with 5 as smallest *or* second anchor’. Given that the coding scheme used to annotate South American and Papuan languages is very similar—it basically revolves around finding anchors and listing them—comparing across areas is straightforward. Analysing compositional anchors in this way serves not only to increase comparability across these two areas, but across all areas, and across both linguistic and non-linguistic systems more generally—especially in systems with limited extent.^3^ Differences that do arise between island New Guinea and lowland South America are pointed out below (see §2a,b and §3a).

This article is explicitly diachronic and uses comparative phylogenetic modelling to estimate ancestral states and test the fit of different models of change. In order to do this, we build on the literature on the diachrony of numeral systems (see §2c; Bowern & Zentz [16], Epps [23], Hurford [24], Owens *et al.* [25], Zhou & Bowern [17]). Specifically, we are interested in the diachronic development of systems with anchors from restricted systems without anchors and in evolutionary ideas about how productive numeral systems are ‘built’ given changes between systems with anchor 2, 5 and 10 (see §2c). We use phylogenetic methods in order to find the evolutionary model that best fits changes between different types of numeral systems as languages change on the branches of their language family trees; these are explained in §3. Results follow in §4 and discussion in §5.

## 2. Numerals in island New Guinea and lowland South America

In this section, we will give a general overview of the literature on numeral systems in Papuan languages in §2a and on numeral systems in South America in §2b. In §2c, we present what we know about diachronic change in numeral systems, building on the literature but mostly concentrating on the two focus areas.

### (a) The Papuan languages of New Guinea

The island of New Guinea is part of a cultural area called Melanesia, which also includes a range of island groups to the east and southeast, notably the Solomon Islands, Vanuatu, New Caledonia and Fiji. On New Guinea, both Austronesian and Papuan languages are spoken. The term ‘Papuan’ is not a genealogical label but rather means ‘non-Austronesian’ and includes more than 70 non-Austronesian families and isolates. While most of the Austronesian languages have reflexes of the ancestral anchor (and possibly base) 10 system [10], Papuan languages are renowned for their restricted numeral systems [25], accompanied by body-based counting systems [26], which in the regional literature are also called ‘body-part’ or ‘digit tallying’ (see §2c below). There is remarkable variety, however, Schapper & Klamer [27] reconstruct what they label a ‘mixed quinary–decimal system’ for Alor-Pantar and in his discussion of rare features of numeral systems, Hammarström [28] mentions Papuan languages with no numerals or with an anchor at 3, 4, 6, and even 15. Here we take Barlow’s [19] paper on Austronesian and Papuan numeral systems as central, as it also constitutes one of the main data sources for the current study. Barlow [19] gathered information about the numeral systems of every Austronesian and Papuan language for which data were available in Chan’s database [29] and from other sources (in total, 1190 Austronesian and 635 Papuan languages). He finds that Austronesian languages outside of Melanesia are almost always decimal, while most of the Papuan languages spoken on New Guinea have a numeral system with an anchor 2 (71% [19, p. 297]), which are limited in their productivity, as these systems often only reach up to 5 or 10.

However, in Melanesia outside of New Guinea, anchor 5 systems abound in both Austronesian languages (57%) and Papuan languages (55%). These anchor 5 systems are believed to originate in Papuan–Austronesian language contact, and Barlow [19] ultimately posits this is true, but not in contact of the type where forms and meanings were borrowed from one language to the other. Rather, given the importance of body-based counting systems in Papuan communities, he posits that Austronesian speakers adopted this cultural practice and newly created ‘quinary’ systems—their inherited decimal systems may have become restricted in usage. He bases this on (i) the areal distribution of ‘quinary’ systems, which are highly prevalent where Austronesian–Papuan contact was most intense; (ii) the lack of reconstructable forms for 3 and 4 in Papuan languages (if these were native, we would expect deep cognate terms); and (iii) low conventionalization (i.e. lack of fixed and frequent sound-meaning combinations) of the Papuan ‘quinary’ systems (and, to some extent, the Austronesian ones). ‘Quinary’ systems emerged 3500 years BP, when the speakers of early East Malayo-Polynesian landed in western New Guinea, and then spread through the descendant Austronesian languages (mostly along the northern coast of New Guinea and surrounding islands) and to Papuan languages through diffusion and contact [19].

### (b) The lowland South American context

Within the continent, Adelaar [30] distinguishes Andean and Amazonian types, with Andean languages having elaborate (but diachronically shallow) systems, mostly decimal, that can go up to 100 000, and Amazonian languages having ‘numeral systems of extreme poverty’ (see also [31]). Both Epps *et al.* [8] and Adelaar [30] mention Pirahã [32], the language that became famous for lacking a numeral system altogether (see electronic supplementary material, section S2b for a similar language from our dataset). Adelaar [30] suggests that languages with Amazonian heritage that are or have been in close contact with Andean languages do develop productive numeral systems; *vice versa*, languages whose speakers move into Amazonia may lose them, related to a cultural rejection of counting. Part of these areal and cultural patterns may be rooted in subsistence style: Epps *et al.* [8] find that languages spoken by hunter–gatherers are more likely to have no terms for numbers that go beyond 5, while agriculturalists are more likely to have numerals that go to 100 and even beyond. They find South America to be home to many restricted and ‘vague’ numeral systems.

Both Epps *et al.* [8] and Epps & Salanova [31, p. 5] comment on the lack of basic numerals in South American languages in general and in Amazonian languages in particular. Basic numerals may be absent altogether, as in Pirahã, and often have primary or additional (vaguer) meanings, such as ‘few’, ‘pair’ or ‘several’. Note that framing these as polysemous/secondary could mean imposing a numeral-centred view—these may be the primary/only meanings and basic numerals may not exist. Even if they are attested, higher numerals may have transparent sources that relate to counting on fingers, other body parts or culturally salient entities that somehow relate to number, for example, the use of the term for ‘brother’, ‘sibling’ in the construction of the word for 4 [31, p. 5]. Zariquiey *et al.* [18] go further and posit that terms in Headwaters Pano languages (Pano-Tacanan) somewhat corresponding to English *one*, *two* and *five* are not true numerals, as they are idiosyncratic, unproductive, inter- and intra-speaker variable and are infrequently used (we return to this in §5a).

### (c) Diachrony

Numeral systems in island New Guinea and lowland South America share two characteristics: in large parts of these areas, restricted systems are the default, while anchor 10 systems are rare. Contact dynamics (very coarsely speaking) seem to differ: according to Barlow [19], 5 as anchor in Melanesia arises through the Austronesian adoption of Papuan body-based counting and its subsequent lexicalization. For South American languages, Adelaar [30] and Zariguiey *et al*. [18] suggest borrowing of form–meaning pairs from contact languages such as Quechua, Aymara, Spanish or Portuguese. A salient area of interaction is found in the Andes lowlands, where Van Gijn and Muysken [33, p. 205] show that (i) elaborate numeral systems are generally present in Andean languages; (ii) native, conventionalized numeral systems that go beyond 9 are attested in several (semi-)lowland languages; (iii) borrowing numeral forms from Aymaran and Quechuan is attested among several families; and (iv) the adoption of Spanish for higher numerals is attested in other lowland languages.

In New Guinea, restricted numeral systems are intimately connected to body-based counting. Barlow [19] classifies languages that construct numerals for 3, 4 and 5 (rarely beyond) by combining the numerals for 1 and 2 (i.e. 2 + 1 = 3; 2 + 2 = 4, 2 + 2 + 1 = 5) as having an anchor 2 system. In Papuan contexts, these anchor-2 systems are used alongside body-based counting systems [25, pp. 41−60], enabling speakers to count beyond 5 using reference to body parts and/or by using body part terms. Indeed, Owens *et al.* [25, p. 285], building on Seidenberg [34], claim that such systems (with an anchor 2 and body-based counting) reflect an ancient state in Papuan languages that has been present for thousands of years. Hurford [24, p. 78ff] considers this situation as a stage in his ideas about the evolution of numeral systems. He likewise emphasizes the importance of tallying systems, where collections of small objects ‘stand in for’ the number of a set of objects. Body-part counting systems do the same using fingers and other body part terms. This early stage may develop into a first language-based numeral system when those body part terms come to represent only or primarily numbers. The next step would be the invention of a system that can be used to create—still simple—compositional numerals through processes including addition, deletion and multiplication. Additional bases and written representations of numerals are still further developments in his overview.

Building on Owens *et al.* [25], we suppose that the Papuan ‘binary’ numeral system plus body-based counting serves as a stable system, sufficient for communal needs for millennia, making the lack of productive numeral systems with an anchor 10 less surprising. In comparison, there is very little study of whether body-based counting typically accompanies numerals or is used to construct higher quantities in South American languages. While anecdotal evidence of counting on fingers and numeral terms reflecting body part terms abounds (see §5a below), body-based counting has not been the topic of comparative study in South America, as it has been for Papuan languages. In this context, Epps & Salanova [31] mention ‘fraternal terms’ where the word for 4 includes the word for sibling or brother, coupled with a paired fingers gesture. Adelaar [30] mentions the ‘rejection of the concept of counting’ in Amazonia—clearly, either the development of a productive numeral system, of body-based counting, or both, must be rooted in favourable economic, social or cognitive conditions, and these should be investigated, especially in South American languages.

So far, we know very little about the interaction between the practice of body-based counting and numeral systems, especially within a diachronic context. Looking at purely verbal numeral systems, it is interesting to consider how the limits of restricted systems with an anchor-2 system may pave the road for anchor 5 and/or anchor 10, and even base 10 systems. Proper binary systems are cognitively not feasible; and even systems using 2 only as minimal anchor, alongside larger anchors, still produce very long number words (see for example Owens *et al.* [25, p. 44ff]) that would become cumbersome if societal interest in counting and other numerical operations increases. Testing whether the shorter forms of numerals in anchor 5 or anchor 10 systems impact diachronic change is thus vital.^4^ However, diachronic studies are very rare—comprising only Epps [23] on the borrowing of numerals into the Amazonian Nadahup family in several stages, Hurford [24], and the work on Pama-Nyungan [16,17]. Importantly, the studies on Pama-Nyungan reveal that languages may lose numerals—which is not that surprising given their often transparent relations with source words (such as body parts) or vague meanings—but also may expand rapidly beyond 5.

## 3. Methods

In this section, we introduce the language sample, data collection and data processing procedures in §3a and the phylogenetic trees we use for the analyses in §3b. In §3c, we detail how the comparative phylogenetic analyses are conducted.

### (a) Data

For the island of New Guinea, we study four language families, Nuclear Trans New Guinea, Nuclear Torricelli, Sepik and Ramu. These contain more than 20 languages, and we have data for more than 20 languages, which is the minimum number for phylogenetic comparative linguistics. Additionally, we carry out analyses of (i) these four families together, called ‘PapuanBig4’, and (ii) analyses for all the families that are found on the main island of New Guinea, excluding Papuan families and isolates of the surrounding islands, called ‘PapuanAll’. In this way we can include far more linguistic data and run a deeper test of the patterns found in individual families. The data come from Barlow’s [19] dataset, yet only on Papuan languages. Table 1 presents the sizes of the datasets and an overview of the phylogenetic tree sets that we used (see §3b).

**Table 1.** Overview of datasets and phylogenetic tree sets. ‘no. lang.’ stands for ‘number of languages’; in the second column, dialects are included in the count, whereas we generally excluded them.

family/ies	no. lang. Glottolog [35]	no. lang. sampled	phylogenetic tree set
nuclear Torricelli	55	43	Glottolog [35]/Dediu (2018) [36]
nuclear Trans New Guinea	316	180	Greenhill (2025) [37]
Ramu	24	21	Glottolog [35]/Dediu [36]
Sepik	36	21	Glottolog [35]/Dediu [36]
*PapuanBig4*	431	289	Bouckaert *et al.* [38]
*PapuanAll*	761	535	Bouckaert *et al.* [38]
Arawakan	77	56	Glottolog [35]/Dediu [36]
Pano-Tacanan	45	31	Glottolog [35]/Dediu [36]
Tucanoan	26	21	Glottolog [35]/Dediu [36]
Tupian	70	63	Glottolog [35]/Dediu [36]

**Figure 1.**
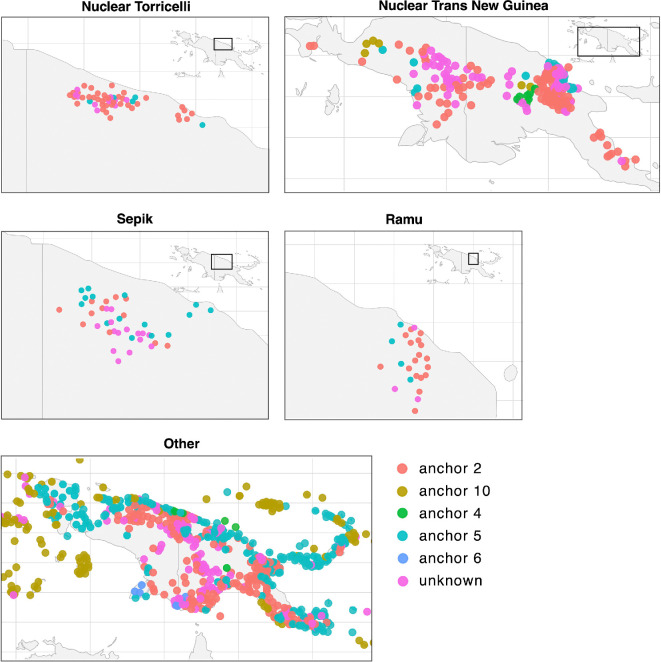
Five-panel map of anchor types across Nuclear Torricelli, Nuclear Trans New Guinea, Ramu, Sepik and other languages of New Guinea island and surrounding islands (the data regarding surrounding islands are not considered in this article).

Barlow’s [19] data were recoded in order to reduce the number of different types of anchors that were attested in certain datasets. Similar categories were merged in order to make the phylogenetic analysis less complex in a sensible way. With each type added, a set of transition rate parameters is added to the model and the number of total transition rates increases exponentially. The more parameters to be estimated during phylogenetic analysis, the harder it is for analyses to converge, making restricting the number of total types a necessary step. Figure 1 shows the areal distribution of the language communities, electronic supplementary material, table S2 gives details on how the data were treated and electronic supplementary material, figures S1–S5, S10 and S11 display the data as they were used as input for the phylogenetic analyses.

**Figure 2.**
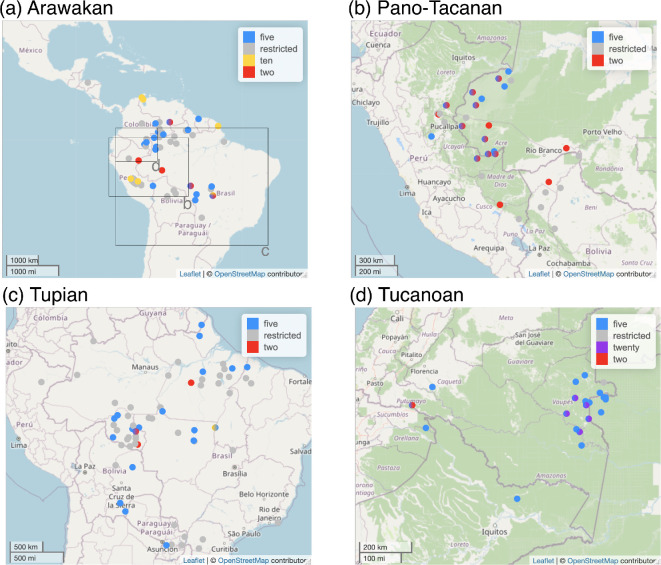
Four-panel map of anchor types across Arawakan, Pano-Tacanan, Tucanoan and Tupian. The boxes in panel (a) refer to the geographical borders of the maps in panels (b–d). Map produced using the R package *lingtypology* [39].

For northern lowland South America, we likewise focused on sizable families and selected Arawakan, Pano-Tacanan, Tucanoan and Tupian—all families with over 20 languages as listed on Glottolog [35]. See figure 2 for their approximate positions and table 1 for an overview of the datasets and phylogenetic tree sets. This leaves several larger and many smaller South American families and isolates unexplored. As there is no equivalent to Barlow’s [19] comparative dataset, a new dataset had to be created by reviewing several existing databases and descriptive literature. The databases are Comrie’s *World Atlas of Language Structures Online* chapter on ‘Numeral Bases’ [6], Grambank with several questions on numeral bases [7] and most importantly Chan’s database on *Numeral Systems of the World’s Languages* [29], as compiled in Numeralbank [40]. These are used to assess evidence for any (or multiple) anchors for all languages of the listed families. Primary literature on languages for which no data were available in any of these three databases is retrieved through Glottolog [35]. These range from complete grammars to word lists in collections. Given the poor description of many South American languages [35,41], some of the collected data are highly tentative. We included data from Ramirez [42] and Kluge [43], two sources that aim to collect information on number words in the languages of the world. It should be noted, though, that in many cases, it remains unclear whether a set of three or four numerals represents the complete set of numerals for that language, leading to a possible bias towards coding such languages as having restricted numeral systems. Furthermore, in most cases, no analysis of the numeral system is offered, so we are quite likely to be misclassifying some systems that have an anchor 2 as unrestricted (see electronic supplementary material, section S2a). Given these caveats, the two abovementioned sources and others did aim to collect full sets of numerals. Although their success may remain tenuous and our dataset tentative, we believe we have constructed a maximally comprehensive resource.

Two specific considerations during data collection were the status of numerals and the treatment of borrowed numerals. Zariquiey *et al.* [18] (current issue) demonstrate that the numeral systems of Headwaters Pano languages do not have proper numerals, as the terms used are poorly conventionalized, rarely used in discourse and the supposed terms for 1 and 2 actually mean ‘single/one/a few’ and ‘pair/two’, respectively. In compiling our dataset, we took numerals described as such at face value and did not account for their (potential) status as terms that are not primarily numerals. Hence, we have coded Headwaters Pano languages as having an anchors 2 and 5.^5^ In future work, we aim to properly code for such differences in status. As for the treatment of borrowed numerals, we aimed to separate information on ‘original’ or ‘native’ systems from that of borrowed material, regardless of its origin. Recent diachronic change, including borrowings, is covered in electronic supplementary material, §S2c. This had the consequence that, for example, we code Shipibo–Conibo as restricted because its decimal system is well-known to have been borrowed from Quechua; however, it has been fully nativized and has been present in the language for a long time. Where to draw the line between ‘native’ and ‘recent’ is, in this respect, an open question where other decisions could have been made.

There are some differences between our newly constructed dataset and that of Barlow [19]. First, in Barlow’s Papuan data, numeral systems are coded by a single anchor (the smallest one). For our dataset, we gather data on all anchors present in a language (see §1). How this impacts our results is discussed below; the coding reflects a difference between Papuan systems—which commonly contain only one anchor and do not go beyond 4—and South American systems, where multiple anchors are often present and the extent of the system is 20. Second, in the South American data collection, we classified languages where the numeral for 2 was clearly involved in the formation of 3 and 4 as ‘anchor 2’; languages with numerals for 1, 2, (3), (4) for which this was not the case were classified as ‘restricted’ because in the majority of cases, those systems did not allow users to count to/beyond 5. Such languages are not attested in Barlow’s [19] Papuan data. Third, Barlow [19] distinguishes between two types of anchor 2 systems: proper and (our term) non-proper. In the first, the anchor 2 term is used to form all subsequent compositional numerals, while in non-proper anchor 2 systems, there is either an atomic form for 3 or for 4 (which is not used as an anchor). We did not code for this detail in the South American data collection.

### (b) Trees

In order to model how numeral anchors change diachronically on the branches of family trees, we need phylogenetic tree sets with branch lengths. We use a variety of these in this article. For Nuclear Trans New Guinea, we use the language family-specific Bayesian tree set from Greenhill [37]. A Bayesian phylogenetic tree set is also available for Arawakan [44], but because it has been criticized by specialists [45], we refrain from using it. For language families where Bayesian tree sets are not available, we are using language family classifications taken from Glottolog [35], with added branch lengths by Dediu [36]. For the PapuanBig4 and PapuanAll analyses, we use parts of a world tree by Bouckaert *et al.* [38]. Preparation of datasets and phylogenetic tree sets as well as plotting of data (see electronic supplementary material, section S4) were done in R [46] using R packages *APE* [47] and *phytools* [48].

### (c) Analysis

We model the type(s) of anchor present in the numeral system of each language in terms of an explicit phylogenetic process, i.e. as the outcome of diachronic processes that take place on the branches of a set phylogenetic family tree (or rather trees, as we incorporate tree samples in order to account for uncertainty in genealogical relations). Of central interest are two aspects of phylogenetic analyses: (i) *ancestral state reconstructions* (probabilistic reconstructions of the behaviour of ancestral languages) and (ii) *transition rate parameters* (simplistically, measurements of how change between anchor types occurs). The former are important because they are central in testing ideas about what types were likely attested in the past, as well as the direction of change. We calculate maximum likelihood estimates for all nodes in the R *corHMM* package [49] for visualization purposes and tentative indicators of diachronic patterns within families. Since these are calculated only on one tree and not on the full tree sample, these do not represent the full picture. We also include formal model testing of the ancestral state of the root (proto-language) in *BayesTraits* v. 4.1.1 [50] by restricting the root to each possible state and comparing log marginal likelihoods (see below). As for transition rate parameters, their presence or absence in the best-fitting models and their estimated values tell us (a) which changes between types are relevant and (b) if there is evidence for any difference in rate (basically the speed of evolution). We will focus on which transition rate parameters are most relevant for explaining the distribution of anchor types within families (and across families for PapuanBig4 and PapuanAll). Related work can be found in [51,52].

A range of different anchor types is in use (for an example, see table 2), ranging from three (Nuclear Torricelli, Ramu and Sepik; see electronic supplementary material, figures S1–S3) up to six (PapuanAll; see electronic supplementary material, figure S11). Hence, we model these using *MultiState* [53] in *BayesTraits* v. 4.1.2 [50]. *MultiState* models character evolution in terms of a continuous-time Markov process (CTM) [53] that describes the probability of change between states of a character (numeral anchors) in terms of a set of ancestral state estimates and transition rate parameters. Each state has several transition rate parameters associated with it: parameters for changes toward that state (and out of any other state) and parameters for changing out of that state (toward all other states). These can be summarized using a transition rate matrix or a *Q matrix*, where each individual transition rate parameter is designated by q, followed by codes for two states. Table 2 illustrates this matrix for the Arawakan language family, in which four anchor types are attested: restricted (coded ‘A’), anchor 2 (‘B’), anchor 5 (‘C’) and anchor 10 (‘D’).

**Table 2.** Q matrix for Arawakan. *trp* (transition rate parameters) stands for transition rate parameters.

type	code	transition rate parameters			
		A	B	C	D
restricted	A	—	qAB	qAC	qAD
anchor 2	B	qBA	—	qBC	qAD
anchor 5	C	qCA	qCB	—	qCD
anchor 10	D	qDA	qDB	qDC	—

Given its four attested anchor types, modelling Arawakan takes 4 * 4 − 4 = 12 transition rate parameters. Since we only have data on 56 Arawakan languages, these are far too many parameters to be estimated. Hence, we use Reverse Jump MCMC (RJ MCMC) [54,55] within *BayesTraits* in order to reduce the size of the evolutionary model. RJ MCMC turns transition rate parameters on and off in an optimal way while at the same time putting the rate estimates in bins shared between parameters. For each individual family, we attempt to come up with a model that fits the data by excluding transition rate parameters that are regularly turned off in the posterior. We run the model without those transition rate parameters and repeat the process in an iterative manner. For the larger families, this implies converging on models with only relevant transition rate parameters left turned on. For the smaller families (Ramu, Sepik, Nuclear Torricelli, Pano-Tacanan), which only have three anchor types attested, this procedure results in a more direct model testing, as there are fewer transition rate parameters. A report on this process, including all output, is included in electronic supplementary material, S5. Aside from a step-by-step turning off of transition rate parameters, we also tested minimal ‘cyclical’ models for each family, in which each listed anchor type can only change into one other. For example, in Arawakan, with four attested anchor types, such a cyclical model implies exclusively the following diachronic changes (only the four associated rate of change parameters would be turned on): (i) restricted > anchor 2 > anchor 5 > anchor 10 > restricted; or its reverse (ii) anchor 10 > anchor 5 > anchor 2 > restricted > anchor 10.

We conduct a single *MultiState* RJ MCMC analysis for each step in exploring the best model for each language family. The number of iterations and the size of the posterior varied given convergence and run time, where we tried to sustain runs to last as long as possible. Convergence was assessed by checking the absence of a correlation between the posterior likelihood and the iteration number; lack of autocorrelation between posterior samples was assessed visually. Reverse Jump was used on all transition rate parameters, with an exponential prior with mean 10. The fit of the model was assessed using *BayesTraits’s* built-in steppingstone sampler, which outputs log marginal likelihoods. These were then compared across runs, with higher log marginal likelihoods indicating a better fit. Subtracting log marginal likelihoods and multiplying the difference by 2 gives us (log) Bayes Factors [50], which we can interpret as ‘positive evidence’ (for > 2), ‘strong evidence’ (for 5−10) or ‘very strong evidence’ (for > 10).

## 4. Results

In this section, we detail the outcomes of modelling choice of compositional anchor on the branches of phylogenetic tree sets. While we do not model geography explicitly, we start by describing areal patterns in anchor choice in each area; results for New Guinea island in §4a are followed by lowland South America in §4b. An important caveat to what follows is that the differences between models are generally very small and not relevant in terms of Bayes’ Factors (as a reminder, BF > 2 constitutes ‘positive evidence’, 5−10 ‘strong evidence’ and >10 ‘very strong evidence’ [50]). This is mostly a statistical power issue: these datasets contain relatively few languages given the number of parameters that have to be estimated (see [52] for discussion). This applies both to the South American results and to those of New Guinea island. Hence, the results that follow should be seen as highlighting the best outcome in a pool of alternative good outcomes.

### (a) New Guinea island

Regarding **areal patterns**, figure 1 shows that anchor 2 systems are used everywhere, whereas anchor 5 systems are found on the northern coast and some way inland. Nuclear Torricelli is spoken in a range of hills close to the northern coast and Ramu to the east of that, with the Sepik family in between. For Nuclear Torricelli and Ramu, there is no geographic pattern in the location of languages with anchor 5 systems. For Sepik, some languages with anchor 5 are found in the north of the area (yet not on the coast): Ak, Awtuw, Pouye, Karawa and Mende. However, other languages with anchor 5 systems are more inland. In Nuclear Trans New Guinea, the situation is different: Anchor 5 systems are found inland (Enga-Kewa-Huli subgroup, Doromu) but more frequently on the coast: the Kaukombaran subgroup (Maia, Mala, Maiani), Western Huon (Komba, Timbe, Selepet, Mese) and the smaller related groups Gum (Amele, Panim) and Garuh-Foran (Nobonob, Wagi) with nearby Matepi and Rempi.

As for **ancestral states**, almost all analyses show (tentative) favour for anchor 2 systems at the proto level (see table 3). For Nuclear Torricelli (BF = 2.0) and Nuclear Trans New Guinea (2.6), this is convincing, for Rama less so (1.6) and for Sepik, all options are almost equally probable (presumably owing to its lack of first-order branching; see electronic supplementary material, figure S3). In terms of visualizing changes on the tree (see electronic supplementary material, figure S1–S5), Nuclear Trans New Guinea, Nuclear Torricelli and Ramu show similar patterns. The majority of the systems present in these languages have a ‘binary’ (proper) anchor, and it seems likely that this was also the case for the proto-language. There are ‘deepish’ changes to anchor 5 and, more infrequently, to ‘binary’ non-proper throughout the tree. By ‘deepish’ we mean that most of these changes concern groups of languages reaching up to 12 members; hence, it is possible that such changes to anchor 5 have taken place in the past, after which descendant languages inherited the anchor 5 system. Sepik, on the other hand, seems to be much messier, with half of the systems having an anchor 5 system scattered throughout.

**Table 3.** Log marginal likelihoods for models where the root state (proto language) was restricted to each attested type per family. Best-performing models are marked in bold, with the supporting Bayes' factor in brackets. The second column concerns systems without compositional anchors, columns 3 through 11, systems with compositional anchors of the indicated value.

family	restricted	2	2 proper	2 n-pr.	5	10	20	4	6
N. Torricelli	—	—	**−39.45** (2.0)	−40.55	−40.46	—	—	—	—
N. Trans New G.	—	**−86.44** (2.6)	—	—	−88.18	−87.72	—	−88.67	—
Ramu	—	—	**−20.39** (1.6)	−21.21	−22.03	—	—	—	—
Sepik	—	—	−24.41	−24.51	**−24.29** (0.2)	—	—	—	—
*PapuanAll*	—	—	**−525.6** (0.5)	−527.67	−526.41	−526.9	—	−525.9	−528.9
*PapuanBig4*	—	—	**−274.2** (1.8)	−275.92	−275.27	−276.09	—	−275.1	—
Arawakan	−60.11	**−58.37** (0.1)	—	—	−59.34	−58.42	—	—	—
Pano-Tanacan	−29.81	−29.73	—	—	**−29.78** (0.1)	—	—	—	—
Tucanoan	−9.52	−9.72	—	—	**−8.43** (2.2)	—	−9.82	—	—
Tupian	**−52.52** (3.8)	−55.57	—	—	−54.43	−56.17	—	—	—

**Figure 3.**
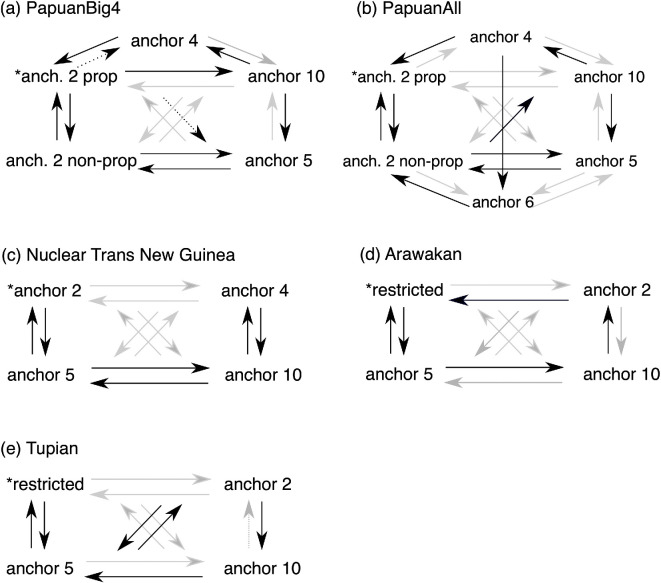
Evolutionary models with the greatest support for five families/analyses. Rate of change parameters in black are turned on; in grey, turned off. These models included a restriction on the root state marked with *. Note: support for these models is not decisive in the majority of cases; see electronic supplementary material, S5. In (a) and (b), not all possible rate of change parameters are drawn, but all were included in the RJMCMC modelling process. Families with small sample sizes are not included.

As for **modelling**, we start with **Nuclear Torricelli** (43 languages), **Ramu** (21) and **Sepik** (21), which are similar in that three anchor types are attested for them (see electronic supplementary material, table S1): ‘binary’ proper, ‘binary’ non-proper and anchor 5. Hence, the analysis started off with six rate of change parameters. In all three families, the process of assessing which parameters were turned off frequently, then removing these and testing further, slightly improved log marginal likelihoods across models; the simplistic cyclical models performed worse (see electronic supplementary material, S5). In terms of Bayes’ Factors, however, it cannot be stated that any model behaves significantly better or worse. This is owing to the small size of these families: There is simply not enough change happening in these families for it to matter how exactly that change is **modelled**. The analysis of **Nuclear Trans New Guinea** is more interesting because of its bigger sample (180 languages). For this family, four anchor types are attested (see electronic supplementary material, table S1). Through assessing model fit by turning off rate parameters, the outcome is a best-fit model with six parameters that performs significantly better than the starting analysis, with no restrictions on the rate of change parameter as well as several others (see electronic supplementary material, S5). As illustrated in figure 3c, the model is symmetrical: change goes from anchor 2 to 5 (and back), from 5 to 10 (and back) and from 10 to 4 (and back). Two alternative models in which change from anchor 4 was only possible out of an anchor 2 system scored significantly worse.

For **PapuanBig4** and **PapuanAll**, the results resemble those of Nuclear Trans New Guinea in the sense that we do arrive at (similar) best-fit models that are significantly better than most of the other models tested (figure 3a,b). An issue that we faced with these analyses is that, while we ran the analyses over Bouckaert *et al*.’s [38] full posterior set of phylogenies, only one or two specific phylogenies are picked every iteration, so these fit best. We tried to find the best-fitting models first, and then force *BayesTraits* to use multiple trees to properly evaluate competing best-fit models. For **PapuanBig4**, we compared two models with nine parameters that perform similarly, and both perform much better than a competitor model with eight parameters (mean BFs are 35.06 and 33.38; see electronic supplementary material, S5). These models only differ on the inclusion/exclusion of two rate of change parameters, marked with dotted lines in figure 3a. In this model, anchor 2 proper, 2 non-proper and 5 are connected in both directions, connecting to a cycle from anchor 2 proper > 10 > 5 > 2 non-proper; with the other anchors in the periphery. For **PapuanAll**, six numeral system types are attested (anchor 2 proper, 2 non-proper, 4, 5, 6 and 10), rendering 30 rate-of-change parameters for the unrestricted model. A model with 10 parameters turned out to be the best supported (figure 3b) in comparison with two competitor models. When we forced *BayesTraits* to use multiple trees to evaluate these three models, support for the 10-parameter model in figure 3b is definite, with a mean BF of 7.64. This model takes change between anchor 2 proper, 2 non-proper and 5 as central: change can take place in both directions in that chain; a cycle appears between anchor 2 non-proper, 10 and 5; and the two rarest anchors, first 4 and then 6, are reached through anchor 10.

### (b) South America

Looking at the four South American families, we can recognize two types: (i) in Arawakan, Pano-Tacanan and Tupian, a substantial number of languages have a restricted numeral system; (ii) in Tucanoan, almost all languages have an anchor 5 system, while some have other anchors. **Areal patterns** are presented in figure 2. Languages with anchor 10 are rare. We find some on the periphery of the Arawakan language family (Palikúr on the Brazilian coast; Paraujano and Wayuu on the Venezuelan coast, Yanesha’ and Yine in Peru and Kustenau in Mato Grosso in Brazil) and Tupian Tapirapé on the border of Mato Grosso province. Restricted numeral systems are found throughout the area, but they are less common in the Vaupés and the area north of it, where we find Tucanoan and Arawakan languages with anchor 5 systems. Anchor 2 systems are less common but seem to be somewhat clustered in the wider area of the Brazilian Acre and southwest Amazonas and across the border in Peru and Bolivia. Interestingly, there does not seem to be an immense effect of contact with Quechua or Aymara, which are major (contact) languages of the Andes with decimal systems (see Van Gijn & Muysken [33]); nor do we observe a clear decrease in restricted systems closer to the Andes versus further removed from them (noting of course that there are many indigenous languages of South America that we do not sample here, so the picture is far from complete).

For **ancestral state estimates**, maximum likelihood estimates are shown on phylogenies for each of the four families in electronic supplementary material, figures S6–S9. Table 3 gives an overview of root-restricted models, in which each attested numeral system type was fixed for the proto-language and support is indicated by the model’s log marginal likelihoods. Together, electronic supplementary material, figures S6–S9 and table 3 provide an impression of the likely system of the proto-language and some tentative changes within the family.

For some families, we find ‘positive evidence’ (when the Bayes’ Factor (BF) is ≥2.0 in comparison with the next best-performing model) for certain numeral systems to be ancestral at the proto-language: Tupian likely had a restricted system and Tucanoan likely had an anchor 5 system. There is no clear outcome for Pano-Tacanan, where all root states seem equally likely, nor for Arawakan, where the proto-language could have been an anchor 2 or an anchor 10 system (BF 0.1). This latter outcome of proto-language system type testing is probably caused by the fact that the few anchor 10 systems in Arawakan all go back quite ‘deeply’ in terms of the phylogeny; this exclusively phylogenetic view would therefore suggest an ancestral anchor 10 system that changed to a restricted or anchor 5 system.

For the modelling, we fine-tuned the *MultiState* RJ MCMC analyses to explore the best-fitting model for each language family. The full results are given in electronic supplementary material, S5. Here, we attempted to find the best-fitting (highest log marginal likelihood) and simplest (as few parameters as possible) models. **Arawakan** (see electronic supplementary material, figure S6 and §S2b-i) has mostly languages with restricted systems, with two subgroups that seem likely to have changed to an anchor 5 system at some point in the past, and with six anchor 10 systems, two of which may have originated in Quechuan contact. For this reason, we model Arawakan with the proto-language constrained to be a restricted system. Its best-performing model is one with five parameters turned on (figure 3d). This model is cyclical, with change between restricted and anchor 5 in both directions, and then a cycle from anchor 5 to 10, to 2 and back to restricted. For **Pano-Tacanan**, we are dealing with severe statistical power issues given that only 31 languages could be coded. The analyses with all possible restrictions on the roots revealed no clear preference (see table 3), and restricting the number of rate of change parameters did not result in a clear best fit (see electronic supplementary material, S5). Proto-**Tucanoan** probably had an anchor 5 system (see table 3) and was modelled with that restriction on the root. The best-scoring models have only four rate of change parameters turned on, either the cyclical set restricted > anchor 2 > anchor 5 > anchor 20 > restricted or its reverse. Again, this is owing to statistical power issues, as we could only sample 21 languages. Proto-**Tupian** likely had a restricted numeral system (see table 3) and was modelled as such. Its best-performing model is one with six parameters turned on; see figure 3e (again noting that a model with five or seven parameters is close in terms of likelihood). This model is also cyclical, with change between restricted and anchor 5 in both directions, and then a cycle from anchor 5 to 2 (connected in both directions), then to 10 and back to 5.

## 5. Discussion

Researching the language families of South America, New Guinea, and possibly Australia and sub-Saharan Africa may be the only chance we have to find out how the world came to be filled with anchor 10 numeral systems: in many families, this trait is ancient, and hence separating community-internal innovation from diffusion is hard. In South America and New Guinea, studying the diachrony of indigenous numeral systems and historical contact-induced change is still possible, and perhaps more importantly, relevant cultural and cognitive perspectives can still be studied. Of course, such research has to take place within its rightful context. The linguistic analysis of numeral systems has focused too little on indigenous counting practices and culture-specific attitudes towards counting and mathematics. In the following sections, we provide tentative generalizations over our quantitative results, first directly for Papuan and South American languages in §5a and then more generally in §5b. In §5c, we give suggestions for further research on South American numeral systems.

### (a) The evolutionary dynamics of anchor choice

What role can phylogenetic methods play in answering questions on numeral system evolution? First, what we have seen here is that taking a genealogical perspective allows us to identify possible or likely changes in the type of numeral system that have taken place in the past; indeed, there is a lot of phylogenetic signal in this data, as is evident from the electronic supplementary material, figures S1–S11. Second, in testing which evolutionary models are most likely to generate the contemporary data, we can assess whether diachronic change is determined by universal or rather local or family-specific pressures (see Dunn *et al.* [56]). **Arawakan and Tupian** have different best-performing models (see figure 3): in Arawakan, a two-way interaction between restricted and anchor 5 systems is central, while in Tupian, change to anchor 2 or anchor 10 systems goes through anchor 5. **Tucanoan** is different yet again, because an anchor 5 system can be reconstructed for this family and overall, not much change is happening. For **Nuclear Trans New Guinea**, we find convincing evidence for the model in figure 3c, which restricts direct changes between anchor 2 and anchor 10—these go through anchor 5 (similar to Tupian). The analyses involving several Papuan language families (**PapuanBig4** and **PapuanAll**) both point to the importance of evolutionary connections between anchor 2 and anchor 5 systems. These findings require further testing, especially from a historical linguistic perspective, to shed more light on (pattern) borrowing, the order of changes and their time-depth. Nevertheless, we may tentatively conclude that we see local (contact-induced change) as well as universal aspects (change from anchor 2 > 5 (>10)) of numeral system evolution.

The (tentative) outcome of our study is that anchor 5 may play a pivotal role in the development of numeral systems, but also that anchor 5 systems (i) may stay stable and having one does not automatically lead to the adoption of an anchor 10 and (ii) are rooted in language contact. The role of anchor 2 is more contentious: in New Guinea, this is possibly the ancestral state, present for millennia and hence the first type of compositional structure present, while for the South American language families, we find that change between restricted and anchor 5 is central, and change from restricted to anchor 2 is far less so. In New Guinea, we find that the languages with anchor 5 systems group together, and the majority of these are spoken on the coast—a situation that is most pronounced in Nuclear Trans New Guinea. Perhaps similarly, we find some potentially diachronically deep changes to anchor 5 in Arawakan and Tupian that may be connected to language contact—here, further research is needed. It would be very interesting to assess whether 5 is somehow favoured as an anchor when initially no anchor or ‘only’ anchor 2 was present, and what role contact with a productive numeral system may play. We hope that our work paves the way for more focused studies of diachronic change of numeral systems in these two areas, with a focus on (i) the reconstruction of numeral forms and counting practices and (ii) modelling contact in a sensible and more direct way, for example, by taking into account movements of speaker communities between Amazonia and the Andes.

Unfortunately, the usefulness of these methods is restricted by the size of language families needed. **Nuclear Torricelli**, **Pano-Tacanan**, **Ramu** and **Sepik** are too small for serious phylogenetic modelling. We have attempted to combine smaller families in the **PapuanAll** and **PapuanBig4** analyses, using a world tree (Bouckaert *et al.* [38]), but are aware that some consider this ‘black magic’, and alternative methods can and should be found (see, for example, Jäger & Wahle [57] or Cathcart & Bickel [58]). However, even with bigger families, model testing has inherent limits, as different models often score similarly in terms of their marginal log-likelihood—there are simply several (sometimes similar) good models of evolution that fit the data. Given the findings on areal convergence, this will be crucial to account for in future work, using qualitative analysis or quantitative approaches (see Ebert *et al.* [59]). A further caveat is that phylogenetic models such as these can only change one state at a time—it would be hard, for example, to account for a change where a restricted numeral system gains an anchor 5 and an anchor 10 at the same time.

### (b) A pathway towards a base 10 system

Compiling the South American dataset, comparing it with the Papuan data and describing the varied results of phylogenetic modelling have led us to a point where evolutionary, diffusionist and cultural aspects may come together to inform a general account of the development of numeral systems. First of all, contact-induced change seems a likely cause for the spread of compositional anchors in these two areas (see also Epps *et al.* [60]). Judging from the maps in figure 2, the Vaupés region and north of it seem to be home to a hub of anchor 5 systems across at least two families, Arawakan and Tucanoan, that are reconstructable to the proto-language for the latter. The origin of decimal systems is probably contact-based for Paraujano, Wayuu, Yine and Yanesha’^6^ (Arawakan); given our focus on ‘native’ systems, we did not code as such other decimal systems where change is known to be contact-induced, such as in Shipibo-Conibo [18], hence historical change to decimal systems is likely underrepresented. The more recent massive shift towards the use of anchor 10 from colonial languages and other lingua francas in South America likewise underscores the importance of contact (see electronic supplementary material, §S2c), as does Barlow’s [19] explanation of the rise of anchor 5 systems in Melanesia. The second pattern we see is one of diachronic stability. The ancestral states attested in Papuan language families reveal a restricted anchor 2 system combined with body-based counting to reach up to 10, 20 or beyond. Similarly, South American families tend to have restricted numeral systems, or at most an anchor 2 or anchor 5 system, whose forms are often rooted in body-part terms. Many of these are unconventionalized terms, or terms that can hardly be called ‘numerals’ (Zariquiey *et al.* [18]). Given that these systems have persisted over millennia, and perhaps only change to accommodate higher anchors and bases ‘in response to’ language contact, we need to take account of the underlying cognitive and cultural factors that drive diachronic change, since contact-induced change is ultimately rooted in socio-historical situations.

In contrast, we have only found limited evidence for the ‘stages’ in Hurford’s [24] diachronic account. In his account, the first use of compositional anchors as we see it in these two areas could form the basis for incipient productive numeral anchors and bases, once body-part terms become number terms. However, the diachronic stability of restricted and anchor 2 and/or 5 systems indicates that a purely linguistic account of increasing system complexity through conventionalization of numeral terms is incomplete. The question is, what triggers such conventionalization and expansion? It may very well be that anchors 2 and 5 are linguistically and practically unusable for more extensive numeral systems and that 10 is a practical and cognitively attractive anchor on which to form a base system, but nevertheless, many languages (or language families) in our two areas only seem to converge on it through contact with other linguistic communities and because of certain social and cultural developments that go hand-in-hand with that contact.

Nevertheless, the sparse anchor 10 systems we find *do* seem to arise from anchor 5 systems, as is the case for Kustenau and Palikúr (Arawakan) and Enga, Enga Wapi and Kyaka (Nuclear Trans New Guinea). For the larger Papuan context, we can also mention Wutung (Sko) and Nafri (Sentanic) on the northern coast of New Guinea. Perhaps these *are* grounded in system-internal expansion—more research is needed. Altogether, for a better understanding of diachronic change in numeral systems, we need to account for changes that may take place within a language or family as rooted in cognitive preferences and cultural predispositions, the effect of contact, as well as non-verbal mathematical practices.

### (c) Future research on South American numeral systems

This article offers a first, tentative look at the evolutionary changes that have been shaping numeral systems. One clear outcome is that the status of numerals in South American families, their roots in body-part terms and the possibility of body-based counting practices have not received enough attention. Overall, Epps *et al.* [8, pp. 70−71], Zariquiey *et al.* [18] and Mello [63] all argue (in different contexts) that South American numerals can be ‘vague’, are ‘unconventionalized’, occur infrequently, or may not be number words at all, as they can be construed as deictic words, or having meanings such as ‘pair/couple’, ‘few’ or ‘another’. We too found that especially Tupian numeral systems have transparent (recent?) lexical origins, terms have multiple meanings, giving the impression of vague and not very well conventionalized numeral systems—but unfortunately, we were not able to code this systematically. Future work on these languages should allow for incorporating these nuances, because they may be decisive for classifying numeral systems as well as for identifying anchors and bases.

The fact that many numerals are polysemous with body-part terms may point towards a possible presence of body-based systems, but should be separated from it. Indeed, many of the terms we encountered appear to be polysemous with body-part terms (we could call these *somatic*)— another dimension for which we did not explicitly code. Placing this in the focus of future work would open the door for much-needed cognacy-based historical linguistic research of these terms.

Another task to address is to collect data on body-part counting practices/systems. We frequently encountered statements on physical gestures that are made during counting or enumeration, pointing to the potential presence of such systems. Future work should try to uncover the relationship between small-scale numeral systems in language (i.e. restricted, anchor 2 and anchor 5 systems) and body-based systems.

## 6. Conclusion

The motivation for this article was to scrutinize numeral systems in regions lacking decimal bases, given that they are so common, so as to shed more light on the diachrony of numeral systems. We focus on two areas—northern lowland South America and the island of New Guinea— where anchor 10 systems are rare and restricted systems of various kinds are the default in large parts of the two areas. Phylogenetic analyses reveal that several of the proto-languages of the studied language families must have had restricted systems or systems with anchor(s) 2 and/or 5, and that these systems can be stable over millennia. Changes to anchor 5 occur in specific subgroups, suggesting that these changes have substantial time depth, and may also be pivotal in later change towards an anchor 10 system. These changes seem to be located in places where ancestral languages were in contact with linguistic communities that (may) have had a different numeral system, illustrating the importance of contact-induced change in numeral system evolution also discussed elsewhere. Further research should investigate in greater detail the impact of counting practices and contact-induced change, while also retaining a diachronic focus by reconstructing numeral forms.

## Data Availability

Data, output and results (S5) can be found on Zenodo: [64]. Supplementary material including section S2-S4 and Figures S1-S9 is available online [65].

## Footnotes

^1^
As detailed in Barlow [3], the linguistic description of systems as quinary or base 5—typically when the numeral for 5 is used as the smallest or a fairly frequent compositional element in constructing larger numerals—is not in line with the concept of base in other disciplines. Here, for conceptual clarity and consistency, we follow the conventions proposed in this theme issue by Pelland [4], and see Chrisomalis [2] and Barlow [3]. However, when referring to linguistic databases and other sources, we will use the original ‘quinary’, yet in simple quotation marks to indicate the difference from proper base 5 systems.

^2^
Here we should point out that one of our main data sources, Barlow [19], uses the terms binary base, quinary system, etc. We map these to our use of the term anchor in electronic supplementary material, table 2 and use the term anchor throughout in order to be consistent, but terms like binary, quinary or decimal may still pop up in quotations or in other appropriate contexts.

^3^
Extent is ‘the largest number expression in a numeration system that can be regularly generated according to its composition rules’; Bender & Beller [22].

^4^
Chrisomalis [5] reports on general patterns in numerical notation, based on extensive investigations of ancient and contemporary systems. He observes the prevalence of anchor 10 systems around the world [5, pp. 515–516] and diachronic change toward decimal in Meso-America [5, p. 553] but does not draw conclusions on this given that anchor 10 systems are so common.

^5^
The only mismatch is Cashinahua (the southernmost language coded as having only an anchor 5 in figure 2b), where we for some reason failed to code for its anchor 2 term correctly.

^6^
For the latter two, language contact with Quechua is a confirmed fact, see Hanson [61, p. 73] on Quechua borrowings in Yine and Adelaar [62] for a detailed study of Quechuan contact effects in Yanesha’.
